# Microarray Analysis in a Cell Death Resistant Glioma Cell Line to Identify Signaling Pathways and Novel Genes Controlling Resistance and Malignancy

**DOI:** 10.3390/cancers3032827

**Published:** 2011-06-27

**Authors:** Janina Seznec, Ulrike Naumann

**Affiliations:** Laboratory of Molecular Neuro-Oncology, Department of General Neurology, Hertie-Institute for Clinical Brain Research and Center Neurology, University of Tuebingen, Otfried-Mueller-Str. 27, Tuebingen 72076, Germany; E-Mail: janina.seznec@medizin.uni-tuebingen.de (J.S.)

**Keywords:** glioma, p53, microarray, IPA

## Abstract

Glioblastoma multiforme (GBM) is a lethal type of cancer mainly resistant to radio- and chemotherapy. Since the tumor suppressor p53 functions as a transcription factor regulating the expression of genes involved in growth inhibition, DNA repair and apoptosis, we previously assessed whether specific differences in the modulation of gene expression are responsible for the anti-tumor properties of a dominant positive p53, chimeric tumor suppressor (CTS)-1. CTS-1 is based on the sequence of p53 and designed to resist various mechanisms of inactivation which limit the activity of p53. To identify CTS-1-regulated cell death-inducing genes, we generated a CTS-1-resistant glioma cell line (229R). We used Affymetrix whole-genome microarray expression analysis to analyze alterations in gene expression and identified a variety of CTS-1 regulated genes involved in cancer-linked processes. 313 genes were differentially expressed in Adeno-CTS-1 (Ad-CTS-1)-infected and 700 genes in uninfected 229R cells compared to matching parental cells. Ingenuity Pathway Analysis (IPA) determined a variety of differentially expressed genes in Ad-CTS-1-infected cells that were members of the intracellular networks with central tumor-involved players such as nuclear factor kappa B (NF-κB), protein kinase B (PKB/AKT) or transforming growth factor beta (TGF-β). Differentially regulated genes include secreted factors as well as intracellular proteins and transcription factors regulating not only cell death, but also processes such as tumor cell motility and immunity. This work gives an overview of the pathways differentially regulated in the resistant *versus* parental glioma cells and might be helpful to identify candidate genes which could serve as targets to develop novel glioma specific therapy strategies.

## 1. Introduction

Glioblastoma multiforme (GBM) is the most common malignant brain tumor. Glioblastomas represent about 60%-70% of all malignant gliomas. Annually in the USA [1] and Europe [2] about three people per 100,000 population develop this most malignant type of brain tumor. Despite the fact that the knowledge about gliomas is continuously growing, the median survival of the patients remains unchanged at 12 months after diagnosis [3,4]. Since these tumors show high resistance against common therapy, there is ample reason to establish new therapeutic approaches in glioma therapy. The tumor suppressor p53 is a transcription factor regulating the expression of genes involved in growth inhibition, DNA repair and apoptosis [5,6]. A dominant positive type of p53, the chimeric tumor suppressor (CTS)-1 is designed to resist various mechanisms of inactivation that are well described for p53 [7]. Adenovirally mediated CTS-1 expression induced growth arrest and loss of viability in human malignant glioma cell lines more efficiently than wild-type p53 [8]. We previously have shown that there are several genes expressed upon CTS-1 overexpression that might be responsible for the superior antitumor properties of CTS-1. Two genes, the quinone oxidoreductase *p53-induced gene 3* (PIG3) and the *putative serine threonine kinase* PCTAIRE3 were strongly induced by an adenovirus encoding CTS-1 (Ad-CTS-1) compared to wild-type p53 [9].

Microarray-based technologies are useful tools to examine gene expression changes in disease and relate these changes to phenotype and clinical data. Recently, several studies on the gene expression in malignant gliomas of different grades have been published [10-12]. In the present study we investigated the changes in gene expression between a parental glioma cell line (229P) and the matched cell line resistant to CTS-1-mediated cell death (229R) using Affymetrix whole-genome microarray expression analysis. The majority of genes differentially expressed in these cell lines were associated within networks that are connected to central tumor-involved players such as *nuclear factor kappa B* (NF-κB), *protein kinase B* (PKB/AKT) or *transforming growth factor beta* (TGF-β). Ingenuity Pathway Analysis (IPA) is a useful tool that supports the understanding of the complexity of the large number of differentially expressed genes that arise from microarray analyses. Here we investigated expression signatures of 229P and 229R cells that might be helpful to identify new target genes most suitable for gene-based therapy of gliomas.

## 2. Results and Discussion

### 2.1. Infection of 229P and 229R Cells with Adenoviral Constructs and Microarray Analysis

**Figure 1.**
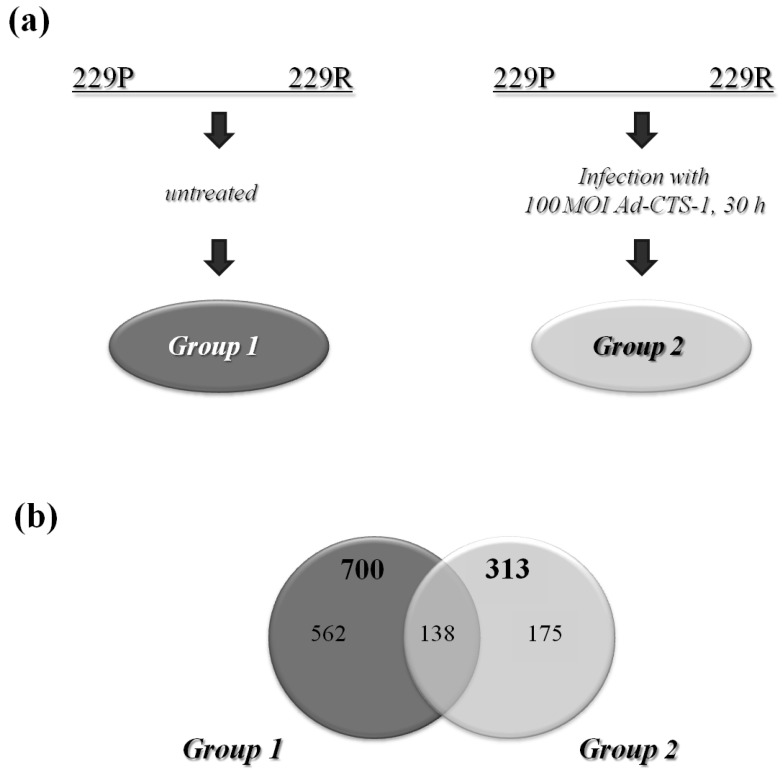
Setting of the whole-genome microarray expression analysis: (**a**). 229P and 229R cells were left untreated (Group 1) or were infected with Ad-CTS-1 (100 MOI, 30 h; Group 2); (**b**). Relationship of numbers of differentially expressed genes in Group 1 (dark-grey) and Group 2 (light-grey). Genes with a fold change of 2.0 or higher and a p-value <0.05 are considered.

In three independent experiments, 229P and 229R cells were infected with Ad-CTS-1 (100 MOI, 30 h) or were left untreated. Thirty hours after infection, the cells were harvested and total RNA was extracted. Whole-genome microarray expression analysis revealed 700 genes that are differentially expressed in the untreated cells (dark-grey; Group 1). In Ad-CTS-1 infected cells, 313 genes are differentially expressed in 229R compared to 229P cells (light-grey; Group 2; Figure 1).

### 2.2. Functional Grouping and Pathway Analysis

*Ingenuity Pathway Analysis* (IPA) software was used to sort the differentially expressed transcripts to functional categories (Table 1). We determined 118 (Group 1) and 86 (Group 2) differentially regulated transcripts that are associated with cancer. The majority of genes are connected to cancer, cell death, cellular growth and proliferation as well as cellular movement.

The most strongly affected transcripts within the microarray analysis are summarized in Table 2.

**Figure 2.**
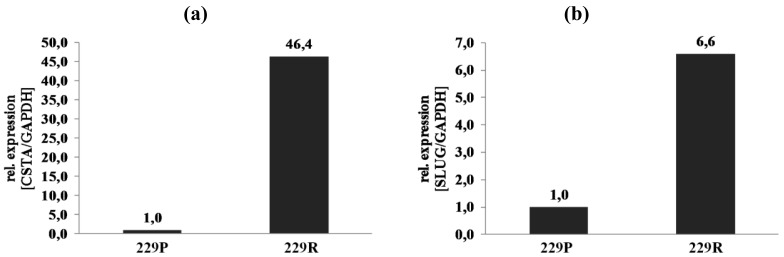
Validation of microarray data by quantitative reverse transcription-PCR (qRT-PCR). Relative expression levels of genes that were differentially expressed in Group 1: (**a**). CSTA, or Group 2; (**b**). Snai2/SLUG in 229P, and 229R cells of samples that were included in the whole-genome microarray expression analysis.

Regulation of the top-regulated transcripts was validated by quantitative reverse transcription-PCR (qRT-PCR). cDNA from RNA of 229P and 229R cells, that underwent microarray expression analysis, was examined (Figure 2, [20] and data not shown).

Figure 2a shows the regulation of *cystatin A* (CSTA). CSTA was 85.05-fold upregulated in 229R compared to 229P cells in the microarray analysis (Group 1). qRT-PCR data confirmed the results whereby expression of CSTA is 46.4-fold upregulated in 229R cells. CSTA plays a crucial role in tumor progression, differentiation, and apoptosis. Induced ectopic overexpression of CSTA rescued tumor cells from TNF-α-induced apoptosis [13]. It therefore might be possible to argue that CSTA could also play a role in CTS-induced cell death of glioma cell lines. *Snail homolog 2* (Snai2/SLUG) is a gene that was shown to be upregulated in Group 2 (upon adenoviral overexpression of CTS-1) in the microarray data. Expression of Snai2/SLUG was 20.69-fold induced in 229R compared with 229P cells (Table 2). In Figure 2b validation of upregulation of Snai2/SLUG post Ad-CTS-1-infection of the cells in the microarray samples has been shown. *Snai2/SLUG* is a p53-regulated gene that is involved in survival, cell spreading, migration, proliferation, and apoptosis. Snai2/SLUG antagonizes p53-mediated apoptosis of hematopoietic progenitors by repressing puma [14], and might therefore play a role in CTS-1-induced cell death. qRT-PCR data confirmed the results of the microarray expression analysis of differentially expressed genes tested so far.

### 2.3. Ingenuity Pathway Analysis

We analyzed the canonical pathways that are affected in both groups and defined a deregulation of different cancer associated pathways linked to NF-κB, PKB/AKT, and TGF-β. Deregulation of these pathways plays an essential role in clinical outcome and survival of GBM patients. TGF-β is a key molecule in glioma mediated immunosuppression, migration and progression [15]. The AKT signaling pathway is frequently constitutively activated by mutations within the tumor suppressor gene *Phosphatase and tensin homolog* (PTEN). AKT signaling mediates proliferation and survival [16]. Inhibitors of NF-κB, PKB/AKT, and TGF-β for treatment of GBM are currently being tested in clinical studies phase I, and II [17-19].

**Figure 3.**
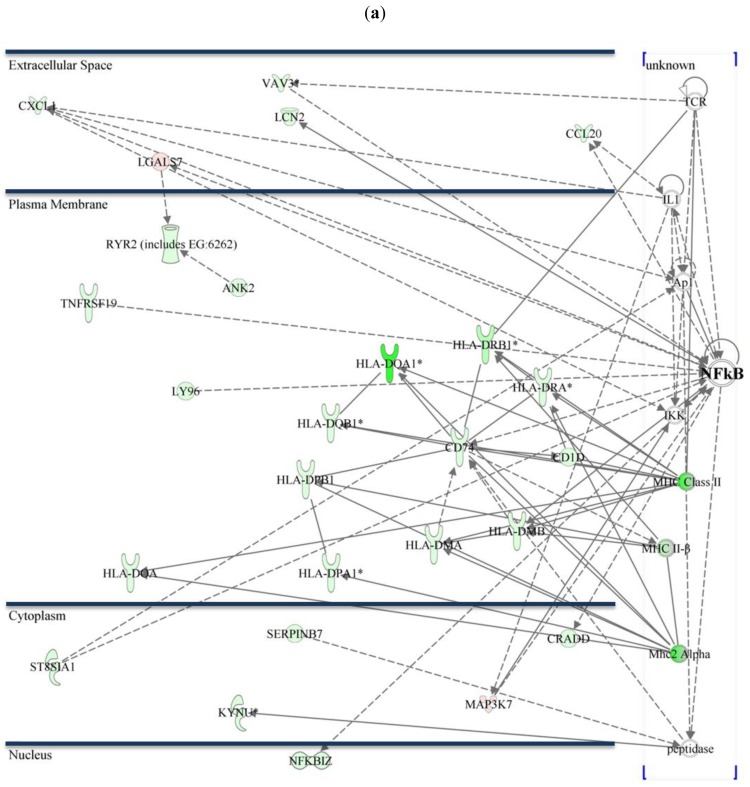

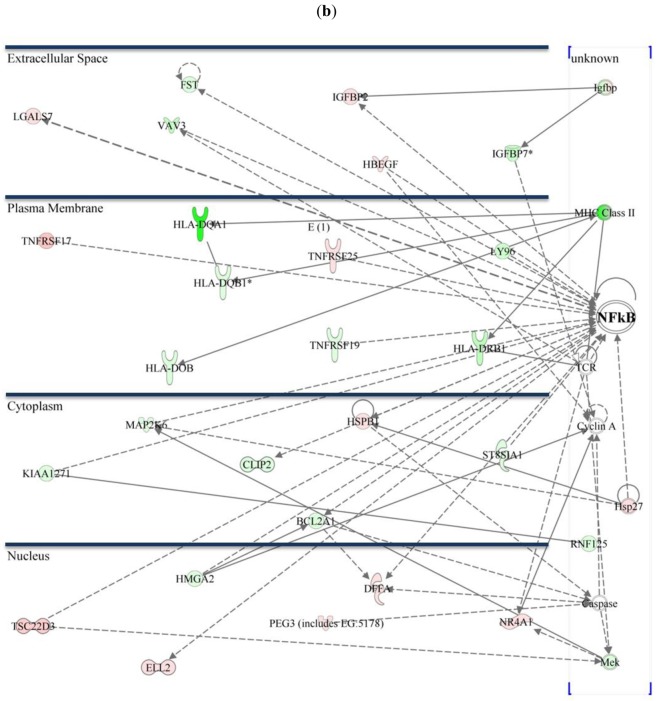
The NF-κB pathway and subcellular localization of differentially regulated genes connected with NF-κB: Within the NF-κB signaling pathway a total of 26 genes were differentially expressed in Group 1 (**a**); whereas 27 genes were shown to be regulated in Group 2 (**b**). (*Green marks* indicate upregulation, *red marks* downregulation of the respective mRNA in 229R cells.)

As previously described, activation of NF-κB plays an essential role in CTS-1 induced cell death in glioma cell lines [20]. Figure 3 shows the NF-κB pathway and figures out the subcellular distribution of regulated genes within the networks in Group 1 and 2. Differentially expressed transcripts that might be interesting for further investigation are chemokines like *chemokine (C-X-C motif) ligand 1* (CXCL1) that plays a role in chemotaxis, migration, and proliferation. CXCL1 confers increased tumorigenicity to glioma cells [21] and is involved in activation of NF-κB and p38 MAPK. *Mitogen-activated protein kinase kinase kinase 7* (MAP3K7), a positive regulator of NF-κB activity, is 2.1-fold downregulated (Group 1) and plays a role in differentiation, cell death, and proliferation [22], whereas *nuclear factor of kappa light polypeptide gene enhancer in B-cells inhibitor, zeta* (NFKBIZ, IκBζ) is a negative regulator of NF-κB [23]. Cell death inducing conditions that are caused via overexpression of CTS-1 lead to induction of genes such as *high mobility group AT-hook 2* (HMGA2; upregulated 28.5-fold in 229R cells, Group 2) that is associated with invasiveness [24], or lead to gene expression such as for *tumor necrosis factor receptor superfamily, member 17* (TNFRSF17) that is 12.05-fold downregulated in 229R cells (Group 2), and is known to increase NF-κB activation in 293 cells [25]. All these genes are involved in the regulation of NF-κB, tumor progression, degree of malignancy and metastasis. Therefore, it is reasonable to conclude that NF-κB may play a crucial role in the development of resistance in malignant glioma.

**Figure 4.**
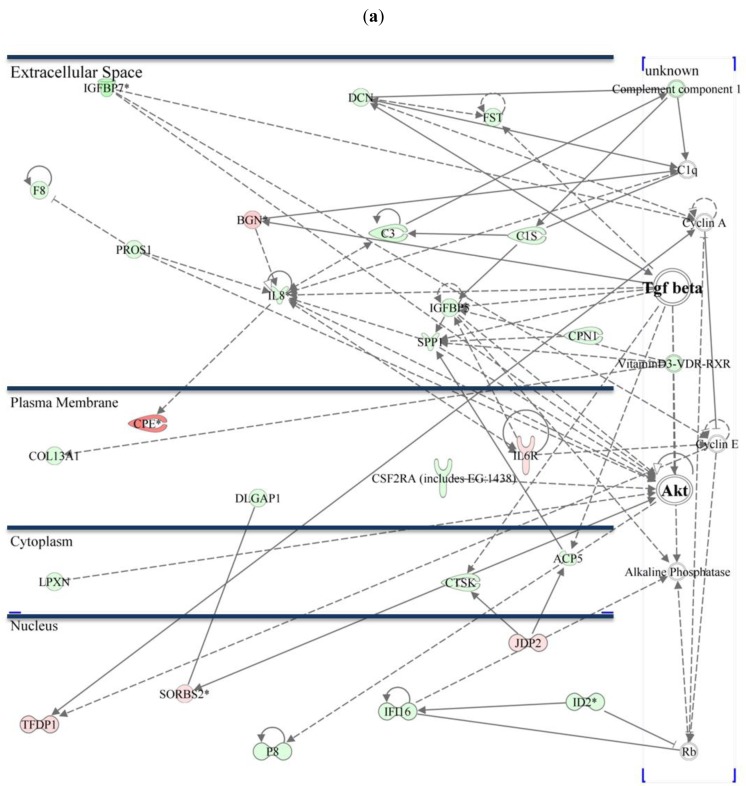

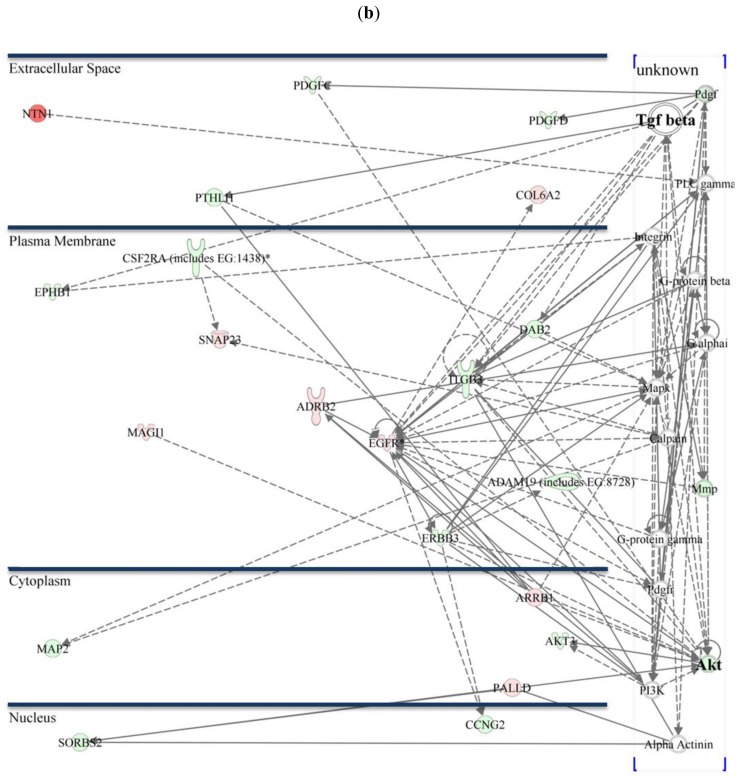
The AKT/TGF-β pathway and subcellular localization of differentially regulated genes connected with this signaling network: Within the AKT/TGF-β signaling pathway a total of 26 genes were differentially expressed in Group 1 (**a**); whereas 21 genes were shown to be regulated in Group 2 (**b**). (*Green marks* indicate upregulation, *red marks* down-regulation of the respective mRNA in 229R cells.)

The AKT/TGF-β (*transforming growth factor beta*) pathway plays an essential role in survival, proliferation, cell death, growth, and migration. Network analysis of differentially expressed genes shows that AKT mRNA expression itself is not differentially regulated in native 229P or 229R cells (Figure 4).

**Figure 5.**
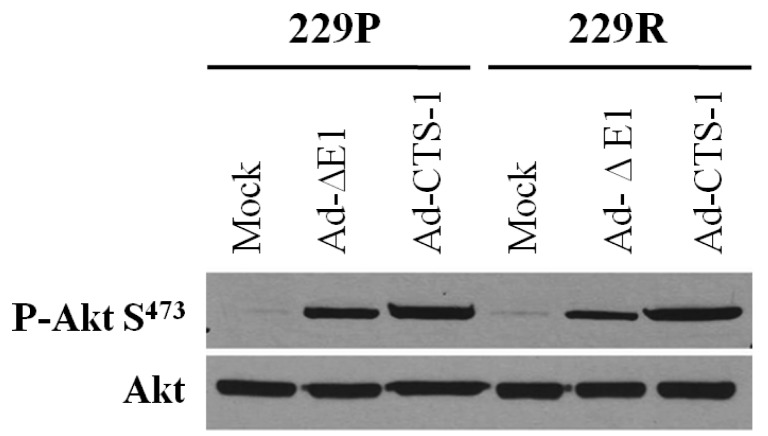
Immunoblot analysis of AKT phosphorylation. The expression of P-AKT (S^473^), and AKT was assessed in untreated (Mock) and adenovirally infected cells (100 MOI Ad-ΔE1 control virus or 100 MOI Ad-CTS-1, 30 h, n = 2).

In our microarray data, overexpression of CTS-1 causes, with a fold change of 1.6, a slight upregulation of AKT mRNA in 229R cells, but AKT protein levels were not significantly changed comparing 229P and 229R cells (Figure 5). AKT itself is phosphorylated upon adenoviral infection; this phosphorylation is further enhanced by CTS-1. Overall, there were no differences in the phosphorylation status of AKT in 229P compared to 229R cells. AKT-phosphorylation is strictly regulated *by phosphatase and tensin homolog* (PTEN) [26]. 229 cells exhibit wildtype PTEN and AKT phosphorylation and activity is strongly regulated in these cells which might explain our observation.

Since the AKT-signaling pathway is intensively cross-linked to TGF-β-signaling, it is rather assumable that TGF-β-signaling associated genes plays a more crucial role in the differences between the two cell lines. The majority of genes that are differentially expressed within the AKT/TGF-β network are TGF-β associated genes. TGF-β is well described to be involved in gliomagenesis. Levels of secreted TGF-β1 and TGF-β2 as well as TGF-β-mediated reporter gene activity are comparable in 229P and 229R cells (data not shown). Nevertheless there are a variety of genes that are directly or indirectly affected by, or were shown to interact with TGF-β.

**Figure 6.**
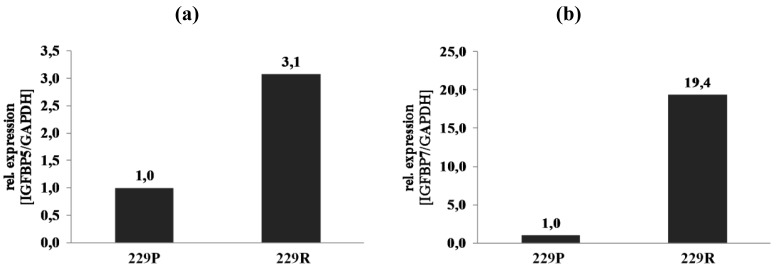
Validation of microarray data by qRT-PCR. Relative expression levels of genes that were differentially expressed involved in the AKT/TGF-β pathway in Group 1: (**a**) IGFBP5 and (**b**) IGFBP7 in 229P, and 229R cells of samples that were included in the whole-genome microarray expression analysis.

*Insulin-like growth factor-binding protein 5* (IGFBP5) is one of the genes that is directly connected to TGF-β in Group 1 and was 4.6-fold upregulated in 229R cells. IGFBP5 overexpression correlates with the histological grade of human diffuse glioma [27]. Microarray data of regulation of IGFBP5 was confirmed by qRT-PCR analysis (Figure 6a). *Insulin-like growth factor-binding protein 7* (IGFBP7) belongs to the group of IGF-binding proteins, is induced in vascularizing GBM [28], and promotes tumorangiogenesis by modulating Smad-2-dependent TGF-β-signaling [29]. Whole-genome microarray expression analysis revealed *IGFPB7* as one of the most strongly regulated genes which was assigned to the AKT/TGF-β network (Table 2, Figure 4a). *IGFBP7* was 25.5-fold upregulated in 229R compared with 229P cells in Group 1, and 9.6-fold upregulated in Group 2. qRT-PCR data confirmed the results of the microarray expression analysis (Figure 6b). IGFBP7 plays a crucial role in growth, proliferation, viability, and angiogenesis [28,29]. In GBM, expression of IGFBP7 correlates with tumor grade, and prognosis [30]. IGFBP7 is a selective biomarker of GBM vessels, induced in and secreted by tumor endothelial cells [29]. In ceratinocytes, IGFBP7 is involved in apoptosis, proliferation, and differentiation [31]. Since the expression of genes involved in differentiation processes is also regulated by IGFBP7, and dedifferentiation is an essential step in tumorigenesis, it might be reasonable to conclude that IGFBP7 has additional functions in the cell lines analyzed here. Up to now there are no studies existing that resolve the role of IGFBP7 in development of resistance in GBM. Further investigation has to be done to clarify the functions of IGFBP7 in this type of cancer [32].

There is a number of genes that might be interesting targets for further investigation: *Colony stimulating factor 2 receptor alpha* (CSF2RA) is involved in the modulation of proliferation and migration of human glioma cells [33]. *Carboxypeptidase E* (CPE), which is highly downregulated in 229R cells (Table 2), seems to be involved in the development or progression of cancer, since in neuroendocrine tumors elevated CPE levels are a significant predictor of good prognosis, and CPE has been described to be a putative prognostic marker in some cancers [34,35]. Binding of *Netrin 1* (NTN1) protein and human p53RDL1 [UNC5B] protein in U373MG cells decreases p53-dependent apoptosis of U373MG cells [36]. *Vav 3 guanine nucleotide exchange factor* (VAV3) mediates the invasive behavior of GBM. VAV3 was 10.87-fold upregulated in 229R cells upon adenoviral overexpression of CTS-1 (Group 2, Table 2), and it is known that downregulation of the guanosine exchange factors, Trio, Ect2, and VAV3 by siRNA suppresses glioblastoma cell migration and invasion in glioma cell lines [37].

Taken together this work gives an overview about genes that might be involved in cell death in 229P, or prevention of cell death in 229R cells. It gives a more detailed insight into regulation of genes in our model of resistance. The refined description of subcellular distribution of regulated targets within the NF-κB, and the AKT/TGF-β pathway might be helpful for a better understanding of this signaling in glioma cell lines. This work facilitates selection of genes that could act as potential targets for innovative glioma gene therapy approaches.

## 3. Experimental

### 3.1. Cell lines and Reagents

LNT-229 (p53 wild-type, polymorphism in codon 72), expressing transcriptional active p53 [38-40] is a human malignant glioma cell line kindly provided by N. de Tribolet (Lausanne, Switzerland). Generation of CTS-1 resistant LNT-229 glioma cells (229R) was previously described [20]. All cell lines were maintained in Dulbeccos modified Eagle's medium (DMEM; GIBCO Life Technologies; Eggenstein, Germany) containing 10% fetal calf serum (FCS), and penicillin (100 U/mL)/streptomycin (100 μg/mL). Ad-CTS-1 was previously described [8]. Resistance to Ad-CTS-1-induced cell death was verified every 3rd passage.

### 3.2. Adenoviral Infection

Infection with recombinant virus was accomplished by exposing cells to adenovirus at a defined moiety of infection (MOI) in serum-free cell culture medium for 15 min followed by addition of serum-containing medium. Viral titers were assessed using a hexon-based titer kit (Clontech, Mountain View, CA, USA). Each virus was tested to be replication deficient. The efficacy of adenoviral gene delivery and expression was ascertained by immunoblot analysis.

### 3.3. Microarray Analysis

229P or 229R cells were infected with Ad-CTS-1 (100 MOI, 30 h) or were left untreated. RNA was isolated using the RNeasy Kit (Qiagen; Hilden, Germany). Quality was checked, 1 μg of RNA was transcribed into double-stranded cDNA and thereon into biotin-labeled cRNA. Microarray analysis was performed as described [41] using the Human Genome U133 Plus 2 genome Array (Affymetrix, High Wycombe, UK) containing more than 54,000 transcripts. Each condition was performed thrice. To constrict for transcripts differentially expressed in 229P *versus* 229R cells, signals were filtered for an absolute change (threshold, Fc) of 2.0. Remaining transcripts were subjected to statistical analysis using a *t*-test. Functional grouping and pathway analysis was performed using the Ingenuity Pathways Analysis software (Ingenuity systems, Redwood City, CA, USA). In this, genes could be sorted to different groups, if their function is known as to be multimodal.

### 3.4. Quantitative Reverse Transcription PCR (qRT-PCR)

229P or 229R cells were infected with Ad-CTS-1 or were left untreated; RNA preparation and qRT-PCR. Isolation of total RNA was performed using the RNeasy Kit (Qiagen, Hilden, Germany). Total RNA was treated with RNase-free DNase I to remove contamination of genomic DNA (Roche, Mannheim, Germany). Five micrograms of total RNA was reverse transcribed using Superscript II reverse transcriptase (Invitrogen, Carlsbad, CA, USA). Expression of the different target genes was determined using SYBR green master mix (Thermo Fisher Scientific, Braunschweig, Germany) on an ABI 7200 detection system. The following primers were used: CSTA-frwd CTGGAGGCTTATCTG-AGGC, CSTA-rev CACCTGCTCGTACCTTAATG; IGFBP5-frwd AAGGACATGCACCGAGAC-CA, IGFBP5-rev CATTGATTGCCACATCCTGG; IGFBP7-frwd CAAGGACATCTGGAATGT-CAC, IGFBP7-rev CCTTTGAACTCCATAGTGACC; SNAI2-frwd CATACCACAACCAGAGAT-CC, SNAI2-rev GAGGAGTATCCGGAAAGAGG; GAPDH-frwd TGCACCACCAACTGCTTAGC, GAPDH-rev GGCATGGACTGTGGTCATGAG. GAPDH primers were used for normalization.

### 3.5. Immunoblot Analysis

Total cellular lysates were generated as described [8]. The following antibodies were used: Anti-Phospho-AKT (S^473^), anti-AKT (all from Cell Signaling, Frankfurt a. M., Germany).

## 4. Conclusions

Our data suggest that a variety of transcripts differentially expressed in our model of resistance to cell death resulted in a wide range of genes that could give interesting new insight in understanding gliomagenesis and progression, and to develop new approaches for glioma (gene) therapy. Our study indicates that expression analysis is a useful tool to investigate genes linked to cancer, malignancy and resistance in therapy. It furthermore potentially enables a better understanding of tumor cell biology, and therefore can be helpful for the development of novel therapeutic strategies. Here we provided further evidence in support of the DNA microarray analysis platform in investigating differentially expressed genes in the pathogenesis of glial neoplasms; in particular, those that are involved in the resistance to cell death. Displaying the major affected targets in a subcellular distribution is helpful to figure out the importance of these proteins within the signaling pathways. To know which gene products, especially gene products that are secreted factors, are involved in cancer or other diseases, is a very helpful tool to analyze whether these secreted factors, which, upon overexpression, could easily be distributed in the tumor mass, are feasible targets for gene therapeutic approaches. Downregulated factors identified in our microarray data might hold tumor suppressor capacity and, if overexpressed, might hold anti-tumor potential. In future, these factors might be used to develop new therapeutic strategies for glioma gene therapy.

## Figures and Tables

**Table 1.** Top bio functions. The large number of genes that are involved in most prominent diseases and disorders or molecular and cellular functions, exhibit the relevance of the regulated genes in the microarray data.

**Group 1**			**Group 2**		

**Diseases and Disorders**	**p-value**	**# Genes**	**Diseases and Disorders**	**p-value**	**# Genes**	

Cancer	6.56E−06–2.37E−02	118	Cancer	4.79E−06–1.66E−02	86
Gastrointestinal Diseases	6.56E−06–2.37E−02	35	Reproductive System Disease	4.79E−06–1.66E−02	38
Connective Tissue Disorders	1.32E−05–4.44E−03	27	Neurological Disease	3.51E−06–1.66E−02	17
Reproductive System Disease	1.96E−05–2.37E−02	85	Dermatological Diseases and Conditions	3.53E−06–1.66E−02	17
Infectious Diseases	1.32E−04–2.32E−02	78	Hematological Diseases	4.96E−06–1.66E−02	12

**Molecular and Cellular Functions**	**p-value**	**# Genes**	**Molecular and Cellular Functions**	**p-value**	**# Genes**

Cell Death	1.32E−05–2.37E−02	81	Cellular Movement	4.79E−06–1.66E−02	40
Cellular Growth and Proliferation	1.96E−05–2.37E−02	82	Cellular Assembly and Organization	1.78E−06–1.66E−02	18
Lipid Metabolism	2.57E−04–2.37E−02	15	Cell Death	4.28E−06–1.66E−02	59
“Small-Molecule“-Biochemistry	2.57E−04–2.37E−02	92	Cellular Growth and Proliferation	5.35E−06–1.66E−02	70
Cell-To-Cell Signaling and Interaction	5.61E−04–2.37E−02	59	Cell-To Cell Signaling and Interaction	8.68E−06–1.66E−02	31

**Table 2.** Top-regulated transcripts. Genes that are regulated in both, non-infected (Group 1) and infected (Group 2) glioma cells, are highlighted (bold).

**Group 1**		**Group 2**	

**Gene**	**Expression Value**	**Gene**	**Expression Value**	

CSTA	85.05	**MAGEA4**	**153.49**
**MAGEA4**	**82.33**	**HLA-DQA1**	**39.94**
**HLA-DQA1**	**75.70**	HMGA2	28.50
C4ORF7	27.67	SNAI2/SLUG	20.69
**HLA-DRB1**	**26.14**	PLA2G7	14.68
**IGFBP7**	**25.52**	**HLA-DRB1**	**11.52**
GDF15	16.59	VAV3	10.87
C3	15.29	**IGFBP7**	**9.63**
TYRP1	13.61	KYNU	8.60
RPS6KA2	11.32	LY96	8.48
**HAPLN1**	−**97.71**	**CPE**	−**120.64**
ABAT	−30.40	**ZFPM2**	−**41.83**
**ZFPM2**	−**23.73**	NTN1	−29.30
**CPE**	−**18.49**	**HAPLN1**	−**27.66**
FHL1	−13.74	FOXP2	−26.79
FAM101B	−11.47	**KCNA2**	−**26.50**
FOXF2	−10.74	DPYSL4	−12.14
**KCNA2**	−**9.82**	TNFRSF17	−12.05
BGN	−7.28	EPB41L4B	−10.51
CKMT1B	−7.22	TSC22D3	−9.87
